# Neuroimmune Pain and Its Manipulation by Pathogens

**DOI:** 10.1111/eva.70098

**Published:** 2025-04-23

**Authors:** Kevin W. Lozo, Athena Aktipis, Joe Alcock

**Affiliations:** ^1^ University of Pittsburgh Medical Center Pittsburgh Pennsylvania USA; ^2^ Department of Psychology Arizona State University Tempe Arizona USA; ^3^ Center for Evolution and Medicine Arizona State University Tempe Arizona USA; ^4^ Department of Emergency Medicine University of New Mexico Albuquerque New Mexico USA

**Keywords:** adaptation, evolutionary arms race, manipulation, nociception, opioids, pain, pain sensitization, pathogen

## Abstract

Recent studies highlight extensive crosstalk that exists between sensory neurons responsible for pain and the immune system. Cutaneous pain neurons detect harmful microbes, recruit immune cells, and produce anticipatory immunity in nearby tissues. These complementary systems generally protect hosts from infections. At the same time, neuroimmune pain is vulnerable to manipulation. Some pathogens evade immunity activated by nociceptors by producing opioid analogs and by interfering with sensory nerve function. Other organisms manipulate neuroimmune pain by increasing it. Hosts may gain protection from interference by adjusting pain sensitivity. Nociceptive sensitization follows expectations of signal detection theory and the smoke detector principle, allowing pain to be more easily triggered in response to microbial threats and damage. However, pain sensitization at the spinal level and cortical responses to pain are themselves the target of manipulation by parasites and other organisms. Here we review examples of parasites, bacteria, and other medically important organisms that interfere with pain signaling and describe their implications for public health, infectious disease, and the treatment of pain.

## 1 Introduction

Pain is estimated to cost the United States economy between $560 and $635 billion (Gaskin and Richard 2012) and is the most common reason patients seek medical care (Debono et al. 2013). Providing pain relief is a priority of health systems and is a worthwhile goal of biomedical research. However, the prevalence of chronic pain is high despite the ready availability of a variety of analgesics (Mansfield et al. 2016). Despite decades of research, the regulatory and feedback mechanisms involved in chronic pain are still not well understood. In this review, we explain the evolutionary dynamics underlying host‐pathogen conflict and how natural selection might favor pathogens that interfere with host pain systems. Hosts, in response, could undergo selective pressure to counter this interference. This can lead to an evolutionary arms race over pain regulation. This perspective provides novel insights for the origin of pain and suggests new solutions for its treatment.

Although pain is said to be the oldest medical problem (Meldrum 2003), it is a double‐edged sword—causing distress for the pain sufferer, but also alerting the body to danger. Nociception, the body's ability to detect potentially painful stimuli, is generally accepted to be functional in the setting of acute pain (Williams 2016). Avoiding or minimizing serious bodily harm is essential for an organism to survive and reproduce (Burrell 2017). The ability to feel and respond to pain is conserved among vertebrates and invertebrates (Burrell 2017), highlighting its protective function (Crook et al. 2014).

When an organism is injured, the response that follows is coordinated by the nervous system, immune system, and endocrine system in an interdependent way (Chapman et al. 2008). This ensemble of responses protects the organism from wounds and other challenges and is part of a general organismal response to stress (Chapman et al. 2008; Nesse and Schulkin 2019).

## 2 The Neuroimmune System—Linking Pain and Immune Function

A wide variety of shared regulatory interactions exist between sensory neurons responsible for pain and effector cells of the immune system (Pinho‐Ribeiro et al. 2017). Pain has long been recognized as a cardinal sign of inflammation, and pain has traditionally been considered to be a downstream consequence of inflammation (Chiu et al. 2012). More recent findings suggest that pain signaling can be the primary event that orchestrates inflammatory responses (Pinho‐Ribeiro et al. 2017). This is perhaps unsurprising, given that the most vulnerable body surfaces are densely innervated, and neuronal signaling offers a rapid way to alert the immune system to danger (Chiu et al. 2012).

Neurons have been shown to drive inflammation independent of classic immune mechanisms. Sensory neurons that are activated by painful stimuli convey information to the central nervous system. At the same time, antidromic axon reflexes, an action potential in the opposite direction, transmit signals to the peripheral tissues which release neuropeptides in nerve terminals (Chiu et al. 2012). These molecules influence immune cell chemotaxis, modify blood flow and capillary permeability, and prime immune cells. Neurons also coordinate immune defenses by detecting the presence of pathogens. Sensory neurons have been shown to express the toll‐like receptor TLR4; stimulation by bacterial endotoxin increases the excitability of these neurons (Liu et al. 2014). These studies highlight a host defense function for the nociceptors responsible for pain and itch (Chiu 2017).

More directly, cutaneous TRPV1 neurons have been shown to detect pathogen molecules and inflammatory cytokines (Cohen et al. 2019) and generate an anticipatory immune reaction in adjacent tissues. These neurons initiated local immune responses against *Candida albicans* and *Staphylococcus aureus* , resulting in an augmented host defense in surrounding tissue distant from the initial site of stimulation (Cohen et al. 2019). This neurogenic inflammation is primarily mediated through substance P and calcitonin gene‐related peptide (CGRP). Chiu et al. (2012) showed that TRPV1 nociceptors in the gut were also shown to detect *Salmonella enterica* . CGRP released by TRPV1 neurons modulated a defense against *S. enterica* infection (Lai et al. 2020). In another example of an anticipatory defense, TRPV1 nociceptors were responsible for an antiviral protein response following skin injury that protects hosts from viral infection, including by herpes simplex virus (Lei et al. 2022).

A recent study illustrates an anti‐helminthic defensive function of mucosal sensory neurons that secrete neuromedin U, a neuropeptide that plays a role in pain perception. Cardoso et al. (2017) showed that the combination of pain‐sensing neurons and immune effector cells provided immediate protection against infection by the helminth *N. brasilensis* . Release of neuromedin U by sensory neurons resulted in the immediate release of inflammatory cytokines by innate lymphoid cells. In this way, neuromedin U was shown to mobilize a mucosal defense against *Nippostrongylus brasiliensis*. Mice lacking the receptor for neuromedin U had a greater infection burden, highlighting the defensive function of this neuroimmune sensory circuit (Cardoso et al. 2017).

**FIGURE 1:**
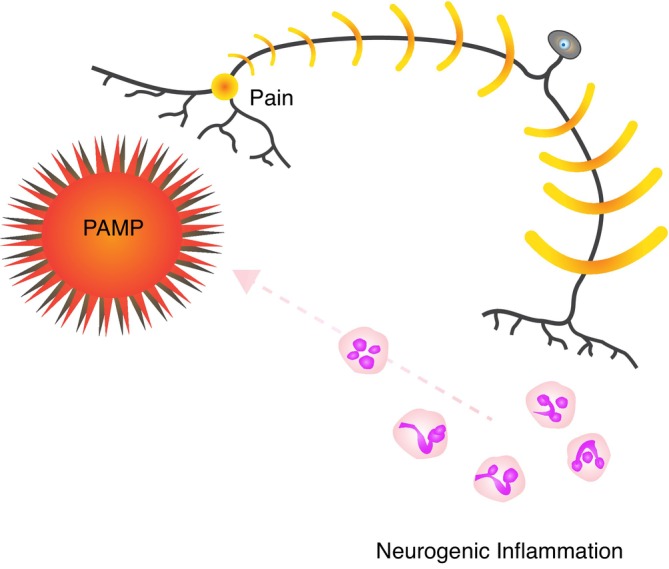
Peripheral nociceptors detect pathogen‐associated molecular patterns (PAMP), (Cohen et al. 2019; Liu et al. 2014). Antidromic axon potentials then release neuropeptides, including substance P and calcitonin gene‐related peptide, that initiate downstream inflammation and provide a host defense against bacterial and fungal pathogens (Chiu et al. 2013; Cohen et al. 2019; Lai et al. 2020).

In these examples, sensory neurons detect microbial signals in a manner that regulates pain and coordinates immune defenses (Figure 1). More broadly, combined functional units, termed neuroimmune sensory units, exist in multiple domains of physiology and have a role in tissue repair and defense (Godinho‐Silva et al. 2019). These integrated systems imply a generally adaptive role of neuroimmune pain, but pain need not always be beneficial for the host. Pain during infection, like other disease manifestations, can represent a defense (one that benefits the host), a manipulation (benefitting the parasite), both, or neither (Ewald 1980). Pain may be an unwelcome non‐adaptive side effect of inflammation. Another possibility is that pain accompanying inflammation is neutral or that pain provides a function to the host unrelated to infection. These options may depend on the specific interaction of the host and parasite. We review the evidence for this in the next section.

## 3 Running Interference—Manipulation of Pain by Medically Important Organisms

Pathogens evolve in competition with their hosts and have a fitness interest in evading detection and immune defenses. In addition to evading immune defenses (Finlay and McFadden 2006) some pathogens hijack the pain system by producing compounds that modulate opioid receptor signaling (Table 1). For example, *E. coli* downregulates pain by producing a toxin that interferes with the opioid receptor system and ultimately counteracts inflammatory pain (Pavone et al. 2009).

**TABLE 1:** Pathogens that interfere with pain and nociception.

Mechanism	Organism	Reference
Inhibits mobilization of TRPV1 receptors to plasma membrane	*Clostridium botulinum*	(Shimizu et al. 2012)
Axon degeneration in sensory neurons and anesthesia	*Mycoplasma leprae* and *Mycobacterium ulcerans*	(Hess and Rambukkana 2019)
Production of opioids or opioid neuropeptides	*Toxoplasma canis*, *Ascaris suum*, *Dracunculus medinensis*, *Schistosoma mansoni, Plasmodium berghei* *Toxoplasma gondii* *Trichninella spiralis*	(Bailey et al. 2000; Pryor and Elizee 2000; Zhu et al. 2002; Golabi et al. 2016; Oyewole et al. 2021; Audibert et al. 2024)
Interference with opioid receptor signaling	*Escherichia coli*	(Pavone et al. 2009)
Synthesis of proteins that mimic enzymes responsible for morphine synthesis in the opium poppy	*Pseudomonas aeruginosa*, *Klebsiella pneumoniae Acinetobacter baumannii*	(Zhan and French 2019)
Metabolic reprogramming, resulting anti‐nociceptive purine metabolites	*Leishmania mexicana*	(Volpedo et al. 2023)
Inhibition of nociceptor TRPA1 by saliva	*Aedes aegypti* (disease vector)	(Cerqueira et al. 2023)
Promotion of pain by alpha hemolysin	*Toxin producing S. aureus*	(Blake et al. 2018).

The parasite *Toxoplasma gondii* is well‐known for manipulating the behavior of its rodent host by making rodents attracted to predator urine. *T. gondii* was recently shown to inhibit nociception in visceral sensory neurons by activating opioid receptors in the gut; this effect was reversed by using the opioid receptor antagonist naloxone (Audibert et al. 2024). Other parasites produce opioid‐like substances, documented, for example, in *Toxocara canis*, an intestinal nematode in dogs that can cause disease in humans (Golabi et al. 2016). The parasitic worm *Ascaris suum* was also shown to contain morphine (Zhu et al. 2004). Additionally, the human parasites *Dracunculus medinensis* and *Schistosoma mansoni* produce a morphine‐like substance with analgesic and immunosuppressive properties (Zhu et al. 2002).

Discovery of opioid‐like molecules in *Schistosoma mansoni* and other parasites suggests that the production of morphine allows these parasites to induce analgesia and evade the host immune system. Opioid‐like neuropeptides showing close sequence similarity to mammalian peptides have been described in trematodes, nematodes, and a leech, suggesting that the strategy of host neuroimmune manipulation might have evolved independently in these phylogenetically distant groups (Pryor and Elizee 2000). Zhan and French hypothesized that similar functions might exist in bacterial pathogens and performed a protein sequence search for T6ODM and CODM, the key enzymes responsible for morphine synthesis in the opium poppy (Zhan and French 2019). This sequence search revealed proteins with a high sequence similarity to T6ODM and CODM encoded by *Pseudomonas aeruginosa* , *Klebsiella pneumoniae* , and *Acinetobacter baumannii* . It is not known yet whether these bacterial sequences have a functional effect that increases virulence or influences host behavior or nociception.

Pathogens interfere with the pain/nociception system by a variety of mechanisms, and some can cause painless inflammation. Some bacteria produce painless lesions, with a well‐known example being the syphilitic chancre caused by *Treponema pallidum* (Chiu 2017). The destructive gum disease periodontitis also involves little pain, despite inflammation. One of the bacterial culprits of periodontitis, *Porphyromonas gingivalis* , inhibits nociception by action of a specific lipopolysaccharide, which also has anti‐inflammatory effects via IL‐10 (Khan et al. 2019). A microbe isolated from dental caries, *Bifidobacteria dentium*, produces GABA that desensitizes nociceptors and has an analgesic effect (Pokusaeva et al. 2017). Many other pathogens also block pain, including *Clostridium botulinum* , *Clostridium tetani* , *Mycobacterium ulcerans* , and *Mycobacterium leprae* (Chiu 2017). *M. ulcerans* produces a polyketide mycolactone that causes hypoalgesia and analgesia by hyperpolarizing neuronal potassium channels (Deng and Chiu 2021). As part of its virulence program, *Mycobacterium leprae* has a unique tropism for Schwann cells of the peripheral nervous system. It directly invades these glial cells and hijacks an intracellular mitogen‐activated protein kinase signaling mechanism that causes axonal degeneration (Hess and Rambukkana 2019). This reprogramming inhibits nerve conduction and confers a survival advantage to the bacterium (Hess and Rambukkana 2019).

Recently, SARS‐CoV‐2 has been shown to block pain via a mechanism of VEGF‐A/NRP‐1 signaling (Moutal et al. 2020). The SARS‐CoV‐2 spike protein hijacks NRP‐1 signaling to reduce VEGF‐A mediated pain. Many viruses, including SARS‐CoV‐2, are known to suppress their host's immune system (Kikkert 2020), e.g., by inhibiting interferon. Evasion of type I interferon via the viral nonstructural proteins NSP 13 and ORF6 may also reduce interferon potentiation of pain during COVID‐19 infection (McFarland et al. 2021). Interestingly, transient hypoalgesia and reduced sensory nerve function has been reported after COVID‐19 infection (Becker et al. 2022; Tereshko et al. 2023). Speculatively, SARS‐CoV‐2's interference with pain during initial stages of infection permits a higher viral load within a host. This reduction in pain is temporary, however, with severe pain often following a period of virus‐induced analgesia.

Herpesviruses have been reported to interfere with pain signaling. The herpes virus HSV‐2 causes variable symptoms ranging from painless lesions to highly painful eruptions, and it also encodes a secreted protein that decreases mobilization of the TRPV1 receptor in the dermis (Cabrera et al. 2016). Early infection of HSV‐1, which has a tropism for the dorsal root ganglion, lowers the excitability of pain‐signaling neurons by interfering with voltage‐gated sodium channels (Zhang et al. 2020). Neuronal reduction in excitability inhibits pain; it also permits increased viral replication (Zhang et al. 2020).

Bites by ectoparasites and hematophagous insects are associated with pain or itch that can initiate defensive immune responses and avoidance behavior in hosts (Chiu 2017). Conversely, some biting insects produce analgesic saliva. *Aedes aegypti* , the mosquito vector of diseases such as dengue fever, has analgesic saliva that interferes with the nociceptor TRPA1 (Cerqueira et al. 2023). The triatomine bugs responsible for Chagas disease have evolved salivary components that inhibit pain, absent in plant‐feeding relatives. Anesthetic saliva might increase the likelihood of an undetected blood meal and the subsequent fecal transmission of *Trypanosoma cruzi* (Zdenek et al. 2024). Saliva of the sandfly *Lutzomyia longipalpis* contains adenosine deaminase enzymes that interfere with nociception during bites (Charlab et al. 2000). This sandfly is the vector for the parasite of cutaneous leishmaniasis, a disease characterized by chronic painless skin lesions (Volpedo et al. 2023). *Leishmania mexicana* alters the metabolism of infected cells, resulting in increased production of purine metabolites with anti‐nociceptive activities. Volpedo et al. (2023) propose that the initial infection and the progression of cutaneous nodules, both of which are painless, modulate host nociception and may thereby promote the spread of infection.

## 4 Host Manipulation by Increasing Pain

A few examples exist of pathogens harnessing the pain system to their advantage by amplifying pain. Blake et al. showed that the Staphylococcal toxin alpha hemolysin increases pain in the process of damaging peripheral pain receptors. This pore‐forming toxin causes calcium influx in sensory neurons, causing an increase in mechanical pain along with thermal pain mediated by TRPV1 sensitization. Increased nociceptor release of CGRP caused by this toxin is associated with impaired bacterial clearance, providing an advantage to the bacterium (Chiu et al. 2013). Although Staphylococcal alpha hemolysin causes intense pain, as opposed to other microbial products that antagonize pain, these results highlight the importance of intact nociception in host defense during infections (Blake et al. 2018).

In addition to alpha hemolysin‐producing *S. aureus* , *Streptococcus pyogenes* also benefits from CGRP release (Pinho‐Ribeiro et al. 2018). *S. pyogenes* secretes the pore‐forming toxin streptolysin S that activates TRPV1 nociceptors. Activation of these neurons releases CGRP and causes severe pain (Pinho‐Ribeiro et al. 2018). In this case, bacteria benefit from immune suppression caused by CGRP release. This has been cited as an example of manipulation by *S. pyogenes* , a pathogen responsible for necrotizing soft tissue infections. As we have described, the pathogen interactions with nociceptors that produce the neuropeptide CGRP have opposing effects on host defense depending on the organism. Detection of pathogens by TRPV1 nociceptors and the release of CGRP generate defensive immunity against *S. enterica* (Lai et al. 2020), methicillin‐resistant *S. aureus* , *C. albicans* (Cohen et al. 2019) and the parasites *N. brasilensis* and *Trypanosoma cruzi* (Borghi et al. 2024).

The nematode *Dracunculus medinensis*, despite its ability to secrete morphine, causes profound pruritis and excruciating pain when the adult gravid worm emerges from the skin. Millions of larvae are then released into the water, where they infect copepods in the next step of the parasite's life cycle. Bakiri and Mingomataj (2010) suggested the itch produced by parasites such as *D. medinensis* might be an effective way to overcome barriers in the skin, effectively a manipulation of their hosts. One might speculate that itching and intense burning pain alter host behavior in a way that benefits the parasite. As Rawla and Jan (2025) write “patients usually place or soak their legs in cold water to get relief from the symptoms, and this causes the worm to break from the blister and emerge from the skin.”

Amplification of pain is a common strategy of venom‐producing invertebrates and vertebrates. A polypeptide component of honeybee venom, melittin, is responsible for most of the pain of a bee sting (Chen et al. 2016). Melittin causes the release of hydrogen ions and inflammatory mediators that open TRPV1 receptor channels on nociceptors and result in pain sensitization. The pain from honeybee and other hymenopteran venoms deters attacks from predators, including *Homo sapiens* , that are many times the size of these insects (Schmidt 2014).

Spitting cobras provide a similar example (Kazandjian et al. 2021). Venom spitting, which has a purely defensive function, is seen in 4000 species of snakes and evolved independently three times. This evolution resulted in increased expression of phospholipase A_2_ toxins that activate TRPV1 sensory neurons in mammals, causing enhanced pain (Kazandjian et al. 2021). Defensive venoms that cause pain have also evolved in at least 2900 fishes (Harris and Jenner 2019). The venom‐sheathed barbs or spines of a stingray or stonefish typically cause long‐lasting pain that is out of proportion to the apparent wound. This pain gives the fish an opportunity to escape to safety. Hyperalgesia caused by a freshwater stingray is a consequence of activation of TRPV1 nociceptors, calcium channels, and release of CGRP (Kimura et al. 2018).

Taken together, these findings support the hypothesis that microbes, helminths, and some invertebrate and vertebrate animals manipulate pain, often in opposing ways. Manipulating nociception may sometimes benefit these organisms while imposing a cost on their human counterparts.

## 5 Opioid Blockade of Pain Can Impair Immune Defenses and Promote Infection

An increasing body of evidence implicates the medical and recreational use of opioids as a risk factor for infection (Plein and Rittner 2018), (Table 2). Opioids have anti‐inflammatory and immunosuppressive effects that are associated with increased susceptibility to bacterial infections. In cell culture and in animal models, morphine impairs leukocyte function and impairs immune defenses, potentially increasing the risk of severe infections (Roy et al. 2011).

**TABLE 2:** Effect of opioid drugs on infection.

Condition	Exposure	Effect	Reference
Abdominopelvic surgery	Preoperative opioid use	Increased postoperative healthcare utilization and morbidity	(Cron et al. 2017)
Hospitalized patients receiving broad spectrum antibiotics	Moderate to high opioid use	Increased risk of *Clostridiales difficile* infection	(Mora et al. 2012)
Patients with and without HIV	Prescription opioid use 12 months prior	Increased risk of community acquired pneumonia	(Edelman et al. 2019)
Invasive pneumococcal disease	Current opioid use	Increased risk of invasive pneumococcal disease	(Wiese et al. 2018)
Rheumatoid arthritis	Current opioid use	Increased risk of hospitalization for infection	(Wiese et al. 2016)
Cirrhosis	Chronic opioid use	Increased risk of endotoxemia, dysbiosis, and readmission	(Acharya et al. 2017)
HIV	Opioid abuse	Accelerated HIV progression	(Meng et al. 2015)
Crohn's disease	Narcotic analgesic treatment	Increased risk of serious infection and mortality	(Lichtenstein et al. 2012)

The immunosuppressive effects of morphine and fentanyl in humans are well documented (Pergolizzi et al. 2008). For example, patients with Crohn's disease receiving opioids had increased mortality (hazard ratio 1.5) and more infections (hazard ratio 3.0) compared with those not receiving opioid analgesics in a longitudinal observational study (Lichtenstein et al. 2012).

Whether these results occur primarily because opioids block pain signaling per se is uncertain. This is because many opioids, including morphine, tend to inhibit immune function, and some opioid users engage in behaviors that increase their exposure to blood‐borne pathogens (Liang et al. 2016; Roy et al. 2011). Along with their anti‐nociceptive effects on sensory neurons, mu opioid receptors are present on white blood cells (Roy et al. 2011). Mu opioid receptor activation on macrophages inhibits phagocytosis and respiratory burst activity and impairs the proliferative capacity of macrophage progenitor cells. Morphine, a mu opioid receptor agonist, interacts directly with neutrophils and dendritic cells and indirectly with natural killer cells via the central nervous system. Short‐term use of morphine has mostly anti‐inflammatory effects that impair responses to infection (Ma et al. 2010; J. Wang et al. 2011). In other words, along with reducing pain, morphine often reduces the effectiveness of the immune response.

Exogenous opioids can have detrimental effects on the microbiome, resulting in dysbiosis in experimental animals (Kang et al. 2017; Mischel et al. 2020; Wang et al. 2018). Morphine caused an expansion of pathogens such as *Enterococcus faecalis* in the gut microbiome of mice (Wang et al. 2018). Opioids have also been shown to increase virulence activation in the gut microbiome of mice (Babrowski et al. 2012) and disruption of intestinal barrier function with translocation of gut bacteria to visceral organs (Meng et al. 2013). In a mouse model of Crohn's disease, opioid‐treated animals showed increased virulence, antibiotic resistance, and toxin production in the gut microbiota; these changes were associated with gut inflammation (Sharma et al. 2020). The increased risk of serious infections in Crohn's disease patients treated with opioids (Lichtenstein et al. 2012) may happen because opioids create favorable conditions for pathogen proliferation.

Since some pathogens benefit from inducing pain, there might be circumstances in which opioids could improve outcomes in infectious disease. Few examples of this exist in the literature. However, naturally occurring endogenous opioids can sometimes boost immune responses (Plein and Rittner 2018). However, a study of naloxone, a drug that abrogates the effects of endogenous opioids, was shown to increase survival and enhance antibody responses to HSV‐1 infection in mice (Jamali et al. 2007).

Some opioids have immune‐enhancing effects that reduce the spread of cancer during surgery (Page 2013). Giving morphine before an experimental surgical stress was shown to preserve natural killer cell activity and to reduce metastases (Page 2013). In a rat model, the opioid buprenorphine showed a better ability to restore natural killer cell function after surgery and with less immunosuppression compared to fentanyl and morphine, partly because of reduced activation of the hypothalamus–pituitary–adrenal axis (Franchi et al. 2007). These results imply that pain itself might sometimes harm immune responses because of the effect of pain on stress hormones. It also suggests that the immune consequences of opioids are drug‐specific and depend on the disease context.

## 6 Pain Plasticity May Augment Host Defenses against Manipulation

Unlike acute pain, which is generally considered to be protective, the conventional view of chronic pain is that it is harmful and maladaptive. The mechanisms of pro‐nociceptive sensitization (i.e., becoming more sensitive to pain) and anti‐nociceptive desensitization (i.e., becoming less sensitive to pain) are involved in chronic pain and its treatment (Benedetti 2008; Crosson et al. 2019; Treede 2016). In vertebrates, the sensitivity of the pain system can be decreased or increased by regulatory events at the peripheral, spinal dorsal root ganglion, and central nervous system levels. Peripheral sensitization occurs after injury by a mechanism of phosphorylation of TRPV1 nociceptors. Sensitized neurons respond with greater output and are triggered at a lower threshold. The dorsal root ganglion is an important site of control over pain sensitization that integrates nociceptive input to the spinal cord. Further regulation over signal processing happens bidirectionally in the brainstem (Treede 2016). In humans, cognitive processes are also able to influence pain, as in the placebo effect (Benedetti 2008). This capacity to regulate pain signal sensitivity and response is broadly phylogenetically conserved. This suggests a functional role for pain sensitization, even though these mechanisms could occasionally produce maladaptive pain (Williams 2016).

Some theorists, including Nesse and Schulkin, have proposed an evolutionary rationale for chronic pain and other aversive symptoms (Nesse 2001; Nesse and Schulkin 2019). They invoke the smoke detector principle–also known as signal detection theory—to explain how apparently excessive responses can be adaptive (Nesse and Schulkin 2019; Nesse and Stearns 2008). With a smoke detector, false alarms are an acceptable price to pay when the cost of not responding to an actual fire is catastrophic. In biological threat detection systems, excessive alarms are the expected outcome of natural selection, giving priority to signal sensitivity over specificity–in other words, ensuring that a real threat isn't missed even if that means overreacting to some false alarms. This smoke detector principle is one of several evolutionary explanations that have been proposed for acute and chronic pain (Table 3). A counter argument to these adaptive explanations is the more conventional view that chronic pain is pathological.

**TABLE 3:** Adaptive explanations for acute and chronic pain.

Hypothesis	Reference
The smoke detector principle	(Nesse and Schulkin 2019)
Protection from further injury	(Wall 1979)
Protection from predation	(Crook et al. 2014)
Increased state of vigilance	(Khuong et al. 2019)
Protection from infection	(Chiu 2017)
Energy conservation	(Watkins and Maier 2000)
Signaling need to conspecifics	(de Williams 2023)

**FIGURE 2:**
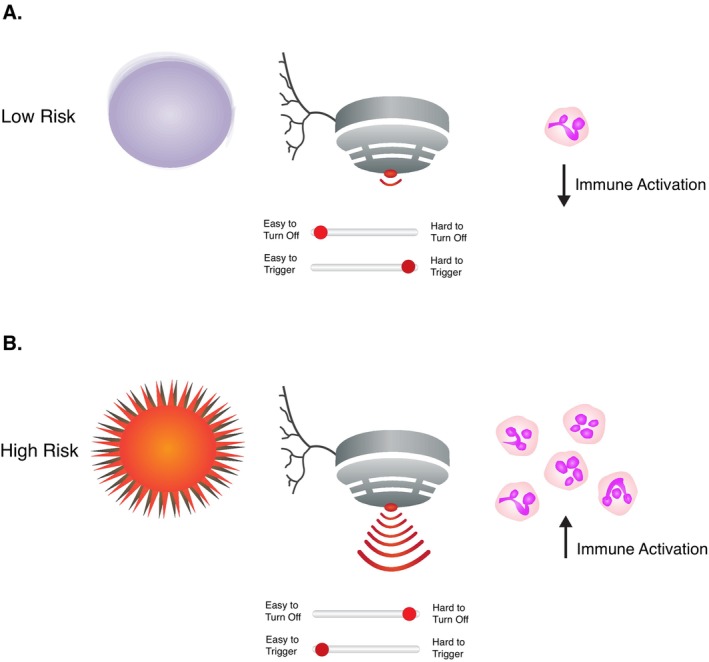
The regulation of host nociception corresponds to the risk posed by pathogens. (A) When the pathogen risk is low, the pain response is hard to trigger and easy to turn off. (B) When the pathogen risk is high, pain “set points” can be recalibrated so that the pain response is easier to trigger and harder to deactivate. Pain triggered by pathogen‐associated molecular patterns is consistent with the regulation of pain by the smoke detector principle (Nesse 2001; Nesse and Schulkin 2019).

As Nesse and Schulkin observed, some chronic pain can occur because too much pain is less costly to the host than too little pain (Nesse and Schulkin 2019). This observation might apply when an organism has a recent experience with infection. Nesse and Schulkin described how repeated encounters with danger would be expected to adjust the “smoke detector” so that the defense is activated at a decreasing threshold (Nesse and Schulkin 2019). Extending this idea, we propose that a lower threshold for triggering defensive pain might be beneficial for hosts when the infection risk is high or when host defenses are impaired (Figure 2).

The mathematics of signal detection theory can be adapted for determining the optimal threshold for pain signaling given the presence of pathogen threats in the environment. As we can see, greater pathogen threats (i.e., higher rates of encounters with pathogens) will reduce the optimal threshold for pain signaling, sensitizing the organism to pain.

$$\text{Threshold for pain signaling}=\frac{\text{Rate of encounters with nonpathogens}}{\text{Rate of encounters with pathogens}}*\frac{\text{Cost of pain signaling if pathogens}\;\mathrm{are}\;\text{not around}}{\text{Cost of not deploying pain signaling when pathogens}\;\mathrm{are}\;\text{around}}$$

In the presence of infection risk, hosts might lower their pain threshold and become–at least temporarily–much more sensitized to pain. When infection threats dissipate, hosts might be less sensitive to pain. This could have led to the evolution of plasticity of host pain signaling and regulation so that it could be adjusted based on changing circumstances. Hosts might also alter pain perception dynamically to compensate for potential manipulation from pathogens.

Del Giudice proposed that the brain and nervous system architecture in humans and other mammals evolved protective mechanisms that anticipate and prevent manipulation by pathogens (Del Giudice 2019). Plasticity in nociception could represent one such counterstrategy.

## 7 Evolutionary Arms Races and Chronic Pain

By manipulating host pain systems, pathogens can gain a fitness advantage, allowing them to expand their population sizes within their hosts. Hosts, on the other hand, are under evolutionary pressure to counter this pathogen manipulation. This co‐evolutionary condition–where one species is evolving to manipulate another and the other species is evolving counter‐manipulation strategies‐ is an evolutionary arms race. And like military arms races, evolutionary arms races can lead to costly escalation. Evolutionary arms races can lead organisms to invest a great deal in conflict, sometimes pushing into physiological space that is risky for all involved parties. Arms races like these can be problematic because they are not just single instances of manipulation and counter response but are instead iterative processes that can end up seriously damaging one or both parties. Some chronic pain may challenge the otherwise functional plasticity of the pain signaling system, especially when hosts are confronted with specialized pathogens that cause a persistent infection and are never cleared by the immune system. Some post‐infectious pain syndromes, e.g., those caused by some herpesviruses, can result from an escalation of conflict that is ultimately disabling for the host.

## 8 The Cognitive Experience of Pain

Although not traditionally considered an emotion, pain accompanies emotional states such as depression and anxiety, and considerable conceptual and mechanistic overlap exists between pain and emotions (Gilam et al. 2020). More generally, emotions and emotion‐valenced states such as pain are proposed to have evolved to solve a survival or reproductive challenge facing an organism (Al‐Shawaf et al. 2016; Cosmides and Tooby 2000). This evolutionary psychology perspective proposes that the internal experience of pain, along with other emotions, can coordinate body and behavioral systems to solve specific adaptive problems. For pain, these problems include protection from injury, conservation of energy, and recuperation from infection.

Evolutionary arguments for pain have centered mainly on protective behavioral changes caused by pain, for instance, protecting an injured limb so that healing can occur (Nesse and Schulkin 2019; Wall 1979). Patients with syndromes of insufficient pain, such as spinal cord dysfunction caused by mechanical injury or infection, fail to perform these protective behaviors and are therefore vulnerable to chronic wounding and joint destruction (Nesse and Schulkin 2019).

After injury, the experience of pain can promote healing behaviors, including temporary loss of use, guarding of injured parts, and grooming (Seymour et al. 2023). Lasselin proposed that the reduction of movement from pain conserves energy and signals sickness to others (Lasselin 2021). In humans, pain‐related signals of need can motivate grooming and helping behaviors (de Williams 2023), promoting caregiving behavior generally (Vigil and Strenth 2014) and in a healthcare setting (Vigil and Alcock 2014). These observations imply that pain reinforces behaviors that promote recovery and proactively reduce exposure to parasites.

The cognitive experience of pain and itch signaling can trigger defensive behaviors that include efforts to avoid biting insects (Chiu 2017; Kupfer and Fessler 2018). Broadly speaking, these avoidance behaviors can be considered part of the behavioral immune system, the set of psychological mechanisms influencing behavior to minimize exposure to pathogens (Amoroso 2021). The regulation of the behavioral immune system by pain is an understudied topic. However, it appears to be vulnerable to manipulation in both directions. Examples include the painless bite of the sandfly vector of leishmaniasis that might help the insect evade defensive swatting or scratching and the sting of a honeybee that discourages honey harvesting and predation.

## 9 Alternative Explanations for Chronic Pain—Nociceptive Sensitization as Protection from Predation

An anti‐infection role of pain we outlined here does not preclude other adaptive functions of pain, such as a heightened state of vigilance (Khuong et al. 2019). Studies involving squid, insects, and rodent models indicate that nociceptive sensitization might protect animals from predators. Crook et al. (2014) showed that injury to the squid *Doryteuthis pealei* resulted in pain sensitization that was associated with the initiation of flight from predators at greater distances and more defensive behaviors such as inking. Without nociceptive sensitization, the injured squid were more likely to be eaten by predators (Crook et al. 2014). Another study of the caterpillar *Manduca sexta* showed that nociceptive sensitization resulted in a decreased tactile threshold for defensive behaviors thought to protect caterpillars from predation (Adamo and McMillan 2019). In *Drosophila*, nerve injury caused the insects to initiate movements away from a thermal stress at a lower temperature and with faster activation of escape muscles, suggesting a heightened state of vigilance (Khuong et al. 2019). In mice, a chronic pain model elicited pain sensitization that resulted in increased aversion to the odor of predator urine, suggesting that pain‐induced vigilance may also be adaptive in mammals (Lister et al. 2020). Unlike the mice studied by Lister et al. (2020), Vyas et al. (2007) showed that rodents infected by *T. gondii* lost their aversion to predator urine. This result implies that anti‐predator vigilance could be a target of manipulation by parasites. It is unknown whether absence of pain sensitization in *T. gondii* infected rodents causes them to lose their fear of predators. However, a recent preprint reported anti‐nociceptive effects in visceral sensory neurons of *T. gondii* infected mice (Audibert et al. 2024).

## 10 Implications for Preventing and Treating Pain

Pain has wide‐ranging effects. In some cases, pain may defend the host from infection. A recent review by Fattori et al. (2021) concludes that nociceptors and immune cells “have evolved to communicate with each other to control inflammatory and host responses against pathogens in a complementary way.” Specifically, peripheral nociceptors release neuropeptides such as CGRP, substance P and neuromedin U that trigger anticipatory immunity in nearby tissues. At the same time, the experience of pain at the cortical level can reinforce behaviors that evolved to protect organisms from illness and infection. Pain sensitization at the dorsal root ganglion and spinal levels may occur in a way that is generally protective. However, just as pathogens have evolved various ways to evade immunity (Finlay and McFadden 2006), some pathogens evolved ways to manipulate pain signaling, either by decreasing or increasing it. Infection by some of those pathogens can result in a chronic pain syndrome, resulting from pain sensitization that is a counter‐adaptation of host or a result of escalating host–parasite conflict.

Potential strategies to manage chronic pain include reducing pathogen exposure, for example by using antimicrobial agents or vaccines. Recently, signaling molecules known as specialized pro‐resolving lipid mediators (SPMs) have been shown to provide antimicrobial effects while also silencing nociceptive signaling (Fattori et al. 2021). Vaccines might control or prevent chronic pain by augmenting immune defenses and preventing or reducing infection. For instance, vaccination can prevent painful outbreaks of shingles caused by the varicella zoster virus. The varicella zoster virus invades cutaneous neurons and travels to the spinal cord dorsal root ganglion where it induces changes in neuronal gene expression along with heightening pain sensitivity (Guedon et al. 2015). There it contributes to the pathophysiology of post‐herpetic neuralgia, a persistent syndrome of disabling chronic pain and heightened sensitivity to pain. Post‐herpetic neuralgia is effectively treated with antiviral therapy (Dworkin et al. 1998) and is prevented with vaccination (Mbinta et al. 2022), suggesting that treating and preventing infections should be considered an important component of chronic pain management.

Alternative analgesic strategies are a priority for chronic opioid users because of the myriad risks of opioids. These include an increased risk of infection and harmful changes to the microbiota. Zhang et al. (2019) showed that morphine caused the depletion of *Lactobacillus* and *Bifidobacteria* in the gut, resulting in pain sensitization and tolerance to the effects of morphine (2019). By causing dysbiosis and subsequent tolerance, opioids can initiate a vicious cycle. Dangerous opioid dose escalation exacerbates dysbiosis and worsens tolerance to opioids and so on (Kang et al. 2017; Mischel et al. 2020). Promisingly, tolerance was mitigated by administering *Lactobacillus* and *Bifidobacteria*, commonly used as probiotics, to mice (Zhang et al. 2019). These results suggest a potential analgesic use for microbes that produce GABA, which has an inhibitory effect on neurotransmission and nociception (Du et al. 2017). The probiotic *Lactobacillus reuteri* has been shown to reduce visceral pain in a preclinical model by inhibiting TRPV1 (Perez‐Burgos et al. 2015). *L. reuteri's* analgesic effects might have first evolved as a manipulation, but this interaction benefits both microbe and host. *L. reuteri* inhibits the growth of gut pathogens and can reverse dysbiosis (Peng et al. 2023). Leveraging the anti‐nociceptive effects of *L. reuteri* has so far had inconsistent results in human clinical trials (Peng et al. 2023).

Counterintuitively, some agents that activate TRPV1 nociceptors are effective analgesics. Ketamine is a dissociative analgesic that potentiates TRPV1 (da Costa et al. 2020). Ketamine has fewer immunosuppressive effects compared to opioids. It also inhibits pathogen growth in vitro (Torres et al. 2018). Capsaicin is another powerful agonist of TRPV1 that has been used for pain relief. After initially activating TRPV1 nociceptors, capsaicin causes a refractory period of decreased TRPV1 sensitivity (Muller et al. 2019). Application of heat temporarily desensitizes TRPV1 nociceptors by a similar mechanism. Immersion in hot water effectively treats severe pain caused by stingray and other marine venoms (Atkinson et al. 2006; Kimura et al. 2018). Considering the defensive role of TRPV1 nociceptors, efforts to develop treatments aimed specifically at blocking TRPV1 should be undertaken with caution. On the other hand, it might make sense to target those nociceptors when provoking pain is a manipulation, as in the case of stingray venom, and for infections by *S. pyogenes* and other bacteria that activate pain as a virulence strategy.

## Conflicts of Interest

The authors declare no conflicts of interest.

## Data Availability

The authors have nothing to report.
